# Supplementary figures and images for: The genomic ancestry, landscape genetics and invasion history of introduced mice in New Zealand
Source: R Soc Open Sci. 2018 Jan 24;5(1):170879. doi: 10.1098/rsos.170879 (PMC5792881; doi:10.1098/rsos.170879)

SF1: Neighbour Joining Tree based on IBS calculated in PLINK, for the GigaMUGA combined dataset.

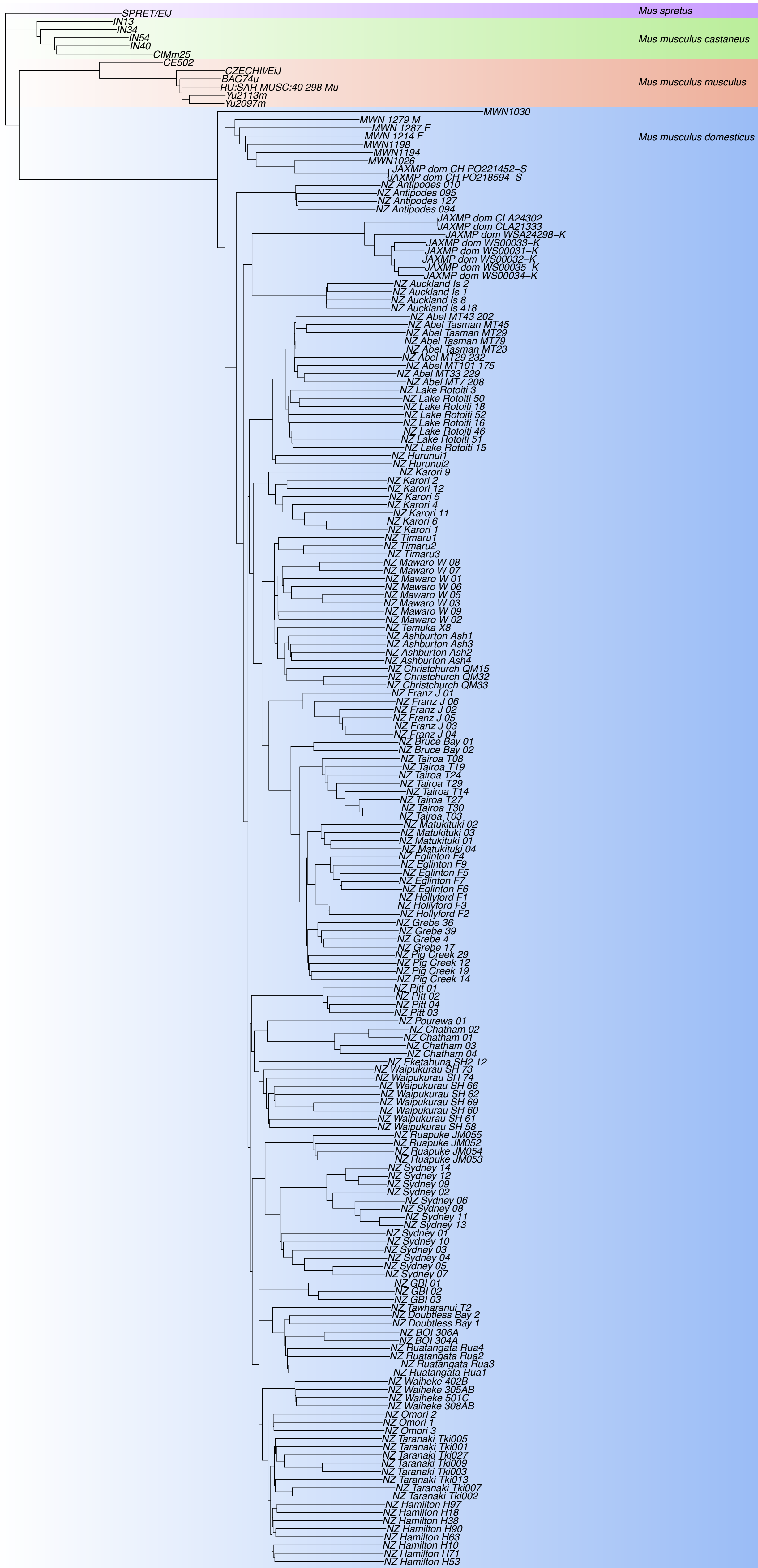

Supplement: Neighbour-joining tree of New Zealand mice with GigaMUGA reference mice [file rsos170879supp1.pdf]

Proportion of 'fixed' SNPs (indicating ancestry proportion)

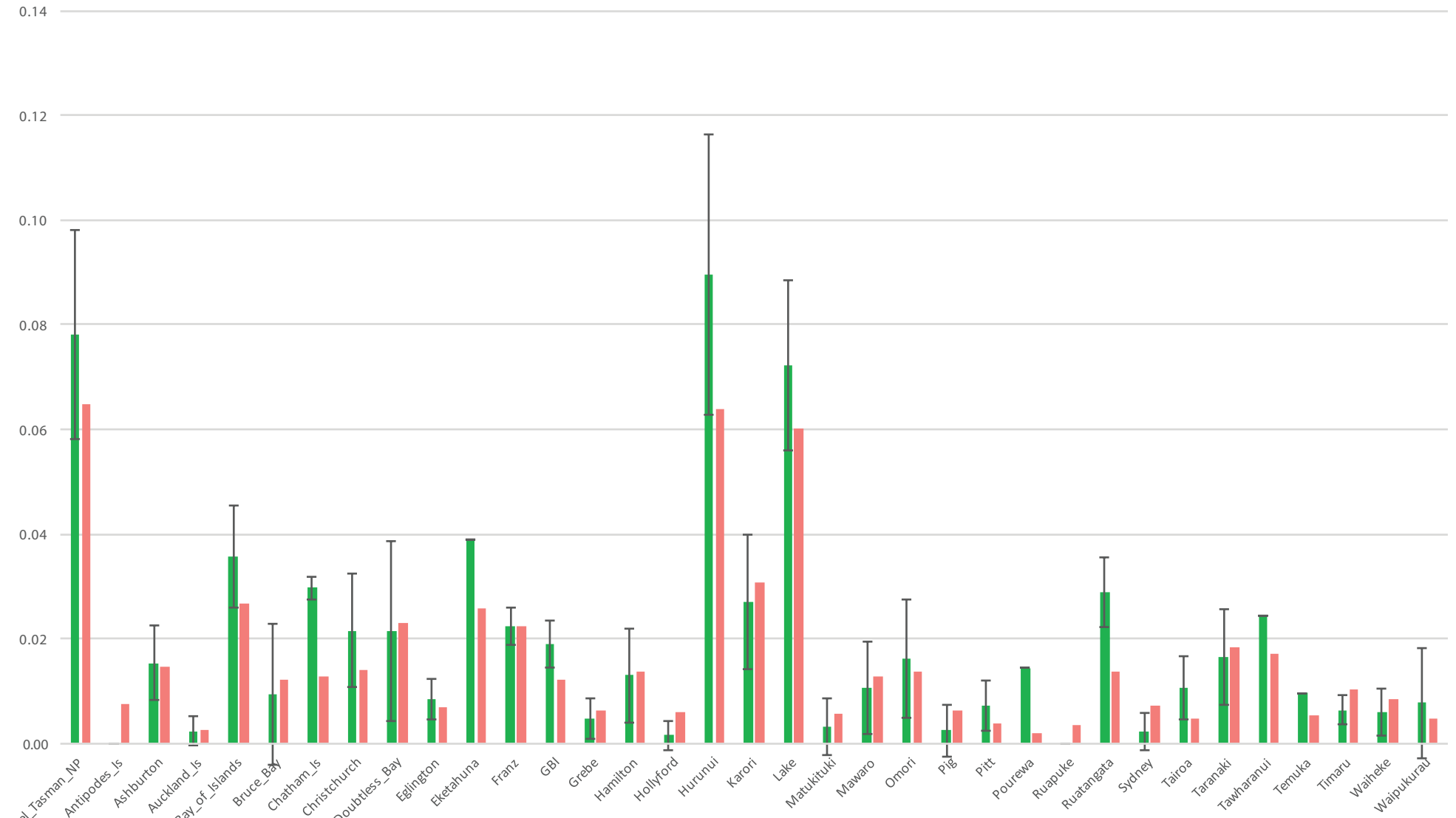

*M. m. musculus*

*M. m. castaneus*

Supplement: SF3: The proportion of 'fixed' SNPs, indicating non-domestics ancestry [file rsos170879supp3.pdf]
