# Supplementary material for: The genomic ancestry, landscape genetics and invasion history of introduced mice in New Zealand
Source: R Soc Open Sci. 2018 Jan 24;5(1):170879. doi: 10.1098/rsos.170879 (PMC5792881; doi:10.1098/rsos.170879)

SF2: Neighbour Joining Tree based on IBS calculated in PLINK, for the MegaMUGA combined dataset.

Country codes (first two letters of ID) are: BE: Belgium, CH: Switzerland, CN: China, CY: Cyprus, DE : Denmark, DK : Germany, EC: Ecuador, ES: Spain, FR: France, GR: Greece, IN: India, IT: Italy, LB: Lebanon, PL: Poland, PT: Portugal, RU: Russia, TN: Tunisia, TW: Taiwan, UK: United Kingdom, US: United States of America

Subspecies highlighted, tree rooted with Mus spretus.

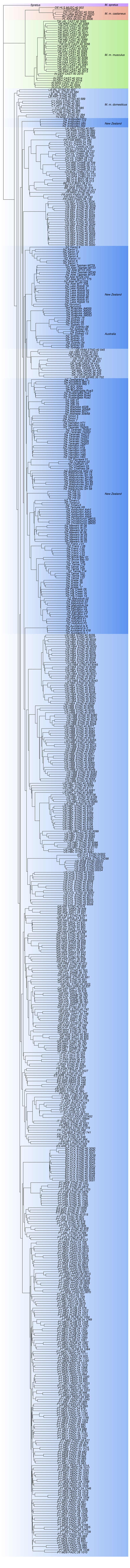

Supplement: Neighbour-joining tree of New Zealand mice with MegaMUGA reference mice [file rsos170879supp2.pdf]
