# Supplementary material for: The genomic ancestry, landscape genetics and invasion history of introduced mice in New Zealand
Source: R Soc Open Sci. 2018 Jan 24;5(1):170879. doi: 10.1098/rsos.170879 (PMC5792881; doi:10.1098/rsos.170879)
Supplement: Supplementary Table ST3 [file rsos170879supp6.docx]

Supplementary Table ST3. Summary of possible agreements between genomic data (this paper) and documentary history (Columns 1-4 reprinted from King 2016) as independent evidence of invasion routes of mice into New Zealand. Columns 3 and 4 list the potential haplotypes found or to be expected in each location, whether or not they have yet been described. Hypothesis 15 added here.

| **Hypothesis #, likely invasion route** | **Approx. date** | ***M. m. domesticus* haplotype** | ***M. m castaneus* haplotype** | **Chances of arrival** | **Agreement with genomic data** |
| --- | --- | --- | --- | --- | --- |
| 1. From Europe with supply fleets (Gabriel et al. 2011) to Sydney | 1788 - 1830 | DomNZ.4 in Sydney |  | Practically certain | Excellent |
| 2a. From India, Batavia with food supplies to Sydney | 1790s-1810s |  | CasNZ.1 in Sydney | Good; Many ships laden with suitable cargoes | None so far |
| 2b. From India to Resolution I., on *Endeavour* wreck (Begg and Begg 1966; McNab 1909:111) | 1795 |  | CasNZ.1 in Dusky Bay | Possible; further testing could identify origin | None so far |
| 3. From Canton with traders returning to Sydney | 1792-1804 |  | CasNZ.1 in Sydney | Unlikely; direct trading on this route restricted by East India Company (EIC) | No *M. castaneus* yet recorded in Sydney. |
| 4. From Sydney with sealers to Fiordland | 1792-1810 | DomNZ.1, 3, 7 south of 44.27S (Canterbury Otago & Southland) | Cas.NZ.1 south of 44.27 S (Canterbury Otago & Southland) | Possible | Possible for domesticus ancestors, but no castaneus mitochondrial haplotypes recorded in Sydney, so this does not explain absence of castaneus on southern offshore islands supplied from Sydney. If both arrived by some roundabout route, hybridisation could have started there. |
| 5. From Canton to Fiordland, avoiding Sydney, with independent sealers’ provisions | 1792-1810 |  | Cas.NZ.1 south of 44.27 S (Canterbury Otago & Southland) | Possible; direct provisioning from China illegal, but profitable until China fur trade no longer economic, c. 1805 (Gibson 1992; Hainsworth 1972; Ross 1987) | Possible location of earliest hybridisation of domesticus-infested ships picking up supplies infested with castaneus in Canton, then landing them in Southland, followed by complete mitochondrial capture of castaneus by domesticus |
| 6a. From Sydney to southern islands with sealers, whalers (Richards 2010) | 1810-30 | DomNZ.2, 5, 8 on Macquarie, Auckland and Antipodes Is |  | High: Frequent sealing/trading voyages between Sydney & subantarctic islands | Good, except for DomNZ.8 on Antipodes, see #15 below |
| 6b. From Sydney to southern islands with sealers, whalers (Richards 2010) | 1810-30 |  | CasNZ.1 on Macquarie, Auckland and Antipodes Is | Very low to zero: furs, whale oil sold in London (Prickett 2002); no return route from SE Asia | None |
| 7. From unknown source via Sydney, on stranded flax trader *Elisabeth Henrietta* | 1824 | DomNZ.19 on Ruapuke Is. |  | Very high: landed with cargo to lighten ship for refloating | Excellent |
| 8. From Sydney to SI south shore sealing/whaling stations, and with settlers to inland Canterbury | 1829-40 | DomNZ.1, 3, 7 south of 44.27S (Canterbury Otago & Southland) |  | Very high: after 1829 frequent trans-Tasman exchanges of whale oil for provisions, livestock (Prickett 2002) | Excellent for domesticus; too late to explain origin of Southland castaneus |
| 9. From China to Dunedin and/or Hokitika with goldminers (Bradshaw 2009; Salmon 1963) | 1865-90 |  | Cas.NZ.1 south of 44.27 S (Canterbury Otago & Southland) | Very likely in Otago, but undetectable if *M. m. domesticus/castaneus* hybrids already present | Undetectable in Otago, but provisioning of Chinese miners to Hokitika direct from Canton is possible explanation for traces of castaneus in Westland. |
| 10. From Europe via Sydney to Bay of Islands (BOI), with traders | 1821 onwards | DomNZ.4 in NZ north of 38.10 S (Northland to Waikato) |  | High: BOI a busy trans-Tasman port from 1820s onwards | Excellent, also, independent visitors from Canton to early Northland offer possible explanation for traces of castaneus there. |
| 11. From Europe, Sydney, to southern NI/northern SI, with traders and settlers | 1840 onwards | DomNZ.1, 3-7, 9-18, 20-21, Mac.domNZ.1-2 from 39.26 S to 44.23 S (Taranaki to Canterbury) |  | Very high: multiple immigrant ships arrived at new ports (500,000 settlers arrived by 1881) (King 2003). | Excellent |
| 12. From China to Wellington and/or Nelson | After 1840 |  | CasNZ.1, musNZ.1, in Wellington and Nelson | Very high: many ships arrived from overseas and local ports | Good, via both international and inter-island trade |
| 13. From SE Asia to Chatham Is. | Unknown |  | CasNZ.2 on Chatham Is. | Unknown | Probably independent, early colonisation followed by complete mitochondrial capture by domesticus from mainland NZ; no known documentary confirmation |
| 14. From Canterbury to Pitt Is. | Unknown | DomNZ.7 (Clade A) on Pitt I., Chathams group |  | Unknown | Excellent |
| 15. Shipwreck | 1908, wreck of *Presidente Felix Faure* | DomNZ.8 on Antipodes |  | Good; all crew and dog landed | Excellent, confirmed French connections of ship with France via New Caledonia |

Bradshaw J (2009) Golden Prospects: Chinese on the West Coast of New Zealand. West Coast Historical and Mechanical Society Inc., Greymouth.

Gabriel SI, Stevens MI, da Luz Mathias M, et al. (2011) Of Mice and ‘Convicts’: Origin of the Australian House Mouse, *Mus musculus*. PLOS One 6:1-6.

Gibson JR (1992) Otter skins, Boston ships, and China goods: the maritime fur trade of the Northwest Coast, 1785-1841. University of Washington Press, Seattle.

Guthrie-Smith H (1969) Tutira. The Story of a New Zealand Sheep Station 4e [1e 1921]. A. H. & A. W. Reed, Wellington.

Hainsworth DR (1972) The Sydney Traders: Simeon Lord and his contemporaries. Cassell Australia, Melbourne.

Jones EP, Searle JB (2015) Differing Y chromosome versus mitochondrial DNA ancestry, phylogeography, and introgression in the house mouse. Biol. J. Linn. Soc. 115:348-361. 10.1111/bij.12522.

King M (2003) The Penguin History of New Zealand. Penguin Books, Auckland.

McNab R (ed) (1908). Historical Records of New Zealand Vol. 1. Government Printer, Wellington.

McNab R (1909) Murihiku (3rd edition): A history of the South Island of New Zealand and the islands adjacent and lying to the south, from 1642 to 1835 Whitcombe & Tombs Ltd 1909, reprinted by Cambridge University Press 2011, Wellington.

Prickett N (2002) The archaeology of New Zealand shore whaling. Department of Conservation, National Historic Heritage Workshop, Wellington.

Richards R (2010) Sealing in the Southern Oceans, 1788-1833. Paremata Press, Wellington.

Ross JOC (1987) William Stewart: Sealing captain, trader and speculator. Roebuck Society, Canberra.

Russell JC (2012) Spatio-temporal patterns of introduced mice and invertebrates on Antipodes Island. Polar Biol. 35:1187-1195. 10.1007/s00300-012-1165-8.

Salmon JHM (1963) A history of goldmining in New Zealand. Government Printer, Wellington.
